# Reseeding Native Species Promotes Community Stability by Improving Species Diversity, Niche, and Interspecific Relationships in the Desert Steppe of Northwest China

**DOI:** 10.1002/ece3.70929

**Published:** 2025-02-05

**Authors:** Haixin Jiang, Yao Zhou, Wen Li, Qi Lu, Dongmei Xu, Hongbin Ma, Xingzhong Ma, Xiaowu Tian

**Affiliations:** ^1^ Research Center for Grassland and Animal Husbandry Engineering Technology of Ningxia Province Ningxia University Yinchuan Ningxia China; ^2^ Key Laboratory for Model Innovation in Forage Production Efficiency, Ministry of Agriculture and Rural Affairs, P.R. China Ningxia University Yinchuan Ningxia China; ^3^ Northern Yanchi Desert Steppe Observation and Research Station of Ningxia Wuzhong Ningxia China; ^4^ College of Forestry and Praticulture Ningxia University Yinchuan Ningxia China; ^5^ Ningxia Zhongning County Forestry and Grassland Bureau Zhongning Ningxia China; ^6^ Ningxia Zhongwei State‐Owned Forestry Farm Zhongwei Ningxia China

**Keywords:** community stability, desert steppe, diversity, interspecific relationships, reseeding native species

## Abstract

The mechanism of community stability is a hot topic in the field of ecology. Research on the stability of the grassland community has gradually increased, and the reseeding of native species is one of the main measures to restore the degraded desert steppe in northwest China. However, little is known about the changes in the stability of the plant community in the desert steppe after reseeding native species. This study established a long‐term observation site for native species reseeding in the desert steppe. We established reseeding and grazing exclusion plots in May 2017 and conducted surveys on degraded grasslands (0YEX(RS)) before setting up reseeding and grazing exclusion treatment experimental plots. After 3 and 6 years of setting up the test, the vegetation restoration status of the plot was investigated, respectively. The results showed that reseeding native species increased the Shannon‐Wiener index and Margalef index of the community. At the same time, the importance value and the breadth of the niche of gramineous plants improved, while the proportion of pairs of high niche overlaps and the logarithm of significant association decreased. The general association of reseeding of the desert steppe was positively correlated, and the stability of the community gradually increased. The results of partial least squares path modelling show that reseeding has a highly significant positive effect on community stability. Both the EX (grazing exclusion grassland) and RS (reseeded grassland) models indicate that niche and diversity indices influence community stability to varying degrees, while interspecific linkage coefficients affect mainly niche overlap. Our research has shown that reseeding native species can improve the intensity of competition between species for resources, leading to a more stable community and ultimately increasing species diversity and community stability. These findings provide valuable theoretical support for vegetation restoration and sustainable management in the desert steppe.

## Graphical abstract

The reseeding of native grass species in the arid steppe of northern China can improve community species diversity, niche, interspecific association and community stability. The results of PLS‐PM path analysis showed that the effect of reseeding on community stability was direct and significant.

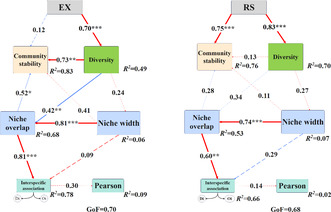

## 1 Introduction

Grasslands are deteriorating due to the combination of global climate change and human disturbance (Cleland et al. 2006; Yue, Shi, et al. 2021). The grassland ecosystem in the arid and semi‐arid areas of northwest China is particularly fragile due to its unique geographical location. As a result, these regions are susceptible to the negative impacts of climate and human activities, which ultimately causes severe degradation of grasslands. The natural process of recovery through succession of degraded grasslands is long (Newbold et al. 2015), but human intervention can help shorten the succession process by removing or controlling the factors that cause grassland degradation (Zhang, Dong, et al. 2020). Previous research has confirmed that reseeding can promote the restoration of grassland vegetation (Samson and Knopf 1994). China began using reseeding to restore degraded grasslands in 1979 (Zhou et al. 2017). Choosing to supplement with nonnative species results in a longer establishment time and creates an unstable community, and supplementing with native species can shorten the time needed for degraded grasslands to recover (Zhang, Xu, et al. 2020), enhancing plant traits and soil quality (Liu et al. 2023; Lu et al. 2023), but the effect of reseeding with native species on the stability of grassland communities is still unclear. The introduction of indigenous grass species has altered the composition and diversity of species of the plant community to some extent, improving the structure and function of the community (He et al. 2021), thus influencing the community succession process. In recent years, the question of the co‐existence of plant communities has become a hot topic in community ecology research (Levine et al. 2017; Zhang et al. 2021; Duan et al. 2023). Scholars often use niche, interspecific association and community stability to reflect the structural characteristics of plant communities and the spatial distribution patterns of plants in their habitats (Rousset and Lepart 2000). Spatial distribution patterns of species can help us interpret species coexistence, the adaptability of community species to their environment, the overlap of ecological niches and the balance of asymmetric competition (Yang and Hui 2021). This can effectively reveal the mechanisms of species succession and community stability (Callaway et al. 2002). Community stability is often affected by biodiversity. However, the influence of ecological niches and interspecies interactions is also of paramount importance. Therefore, it is necessary to quantify the interspecific relationships through the ecological niche and the associated characteristics of species.

Plant ecological niches and species interactions are two hot topics in ecology research. Ecological niche refers to the ability of a species to obtain resources, cooperate and compete in a given environmental space (Zhao et al. 2018). Ecological niche characteristics are often quantified using niche width and niche overlap (Laaka‐Lindberg, Pohjamo, and Korpelainen 2005; Martinez et al. 2010; Giustino et al. 2018). Community species interactions, or the degree of association between species, can effectively demonstrate their spatial distribution at the individual species level (Martinez et al. 2010). This reflects a species' adaptation to the environment and is a comprehensive representation of species interactions and impacts, determining community composition, structure, function and dynamic changes (Du et al. 2024). It is the basis for the formation and evolution of communities. By describing the position occupied by populations or individuals in a community, we can identify community development and succession trends and understand the competitive exclusion effects between species, allowing for effective human intervention.

Human intervention in grasslands over time has led to an increase in neutral interactions between species, resulting in a decrease in the number of noticeable positive and negative associations within the community (Wu, Cheng, and Liu 2022). The complexity of species composition has a significant impact on community stability (Niu et al. 2018). The composition of dominant species and species diversity typically determine the future development of the community (Gross, Cardinale, and Fox 2014; Zhang et al. 2024). Understanding changes in plant community structure and species composition and predicting future succession processes through interspecific interactions is crucial for the restoration of degraded grasslands (Hautier et al. 2015; Zhang et al. 2016). Currently, the research focus on niche and interspecific relationships is concentrated in temperate grasslands, tropical rainforests and alpine meadows (Lanszki et al. 2019; Juan et al. 2023). The research direction focusses mainly on different habitat types, fertiliser addition and different degradation gradients (Juan et al. 2023; Du et al. 2024). Scholars have mostly studied desert steppe reseeding from the perspectives of soil microbial communities (Zhang, Zhou, et al. 2020), community productivity (Guo et al. 2018; Yue, Zuo, et al. 2021) and evaluation of restoration effectiveness (Chen, Weber, and Schnase 2012; Duan et al. 2023). However, there is limited research on the effects after reseeding on interspecific relationships in grassland plant communities, especially the response strategies of native species reseeding to the species niche and association mechanisms in the desert steppe and their impact on diversity and community stability are still unknown.

The desert steppe is the most arid type of grassland vegetation in the transition from grassland to desert, and the ecosystem is extremely fragile (Xu et al. 2024). Due to overgrazing by herders, varying degrees of degradation have occurred in this ecosystem (Hou, Sun, and Wang 2018). China has implemented various ecological restoration measures to restore degraded grasslands, achieving significant results. The study found that various vegetation restoration measures have many factors that affect plant communities. Differences in the distribution patterns of dominant species can also lead to different restoration effects. It is vital to elucidate the co‐existence and cooperation between native species and reseeded species, and the selection of grass species for reseeding plays a key role. Reseeding native grass species has become the preferred method of restoring degraded desert steppe due to their strong adaptability. We selected the semi‐arid desert steppe of Ningxia as our research subject. By investigating the species composition of long‐term plant communities in native desert steppe reseeded with native species and combining with the pretreatment conditions of degraded desert steppe restoration, we used the community combination effect of plant community species niche and interspecific correlation to explore the impact of native grass species reseeding and enclosure on the structure and stability of desert steppe plant communities. This is expected to provide data and theoretical support for vegetation restoration in the desert steppe.

To explore how native grass species respond to reseeding in degraded desert steppe, this study examined the characteristics of the plant community of reseeded areas 3 and 6 years after reseeding while also comparing them with protected and degraded sites from the same period before restoration efforts. This research aims to address the following scientific enquiries: (1) In what way do the stability and species diversity of plant communities on degraded desert steppe evolve with the passage of time after reseeding native species? (2) Can the connection between species ecological niches and interspecies interactions to some extent affect the changes in community species diversity and stability?

## 2 Materials and Methods

### 2.1 Study Site

**FIGURE 1:**
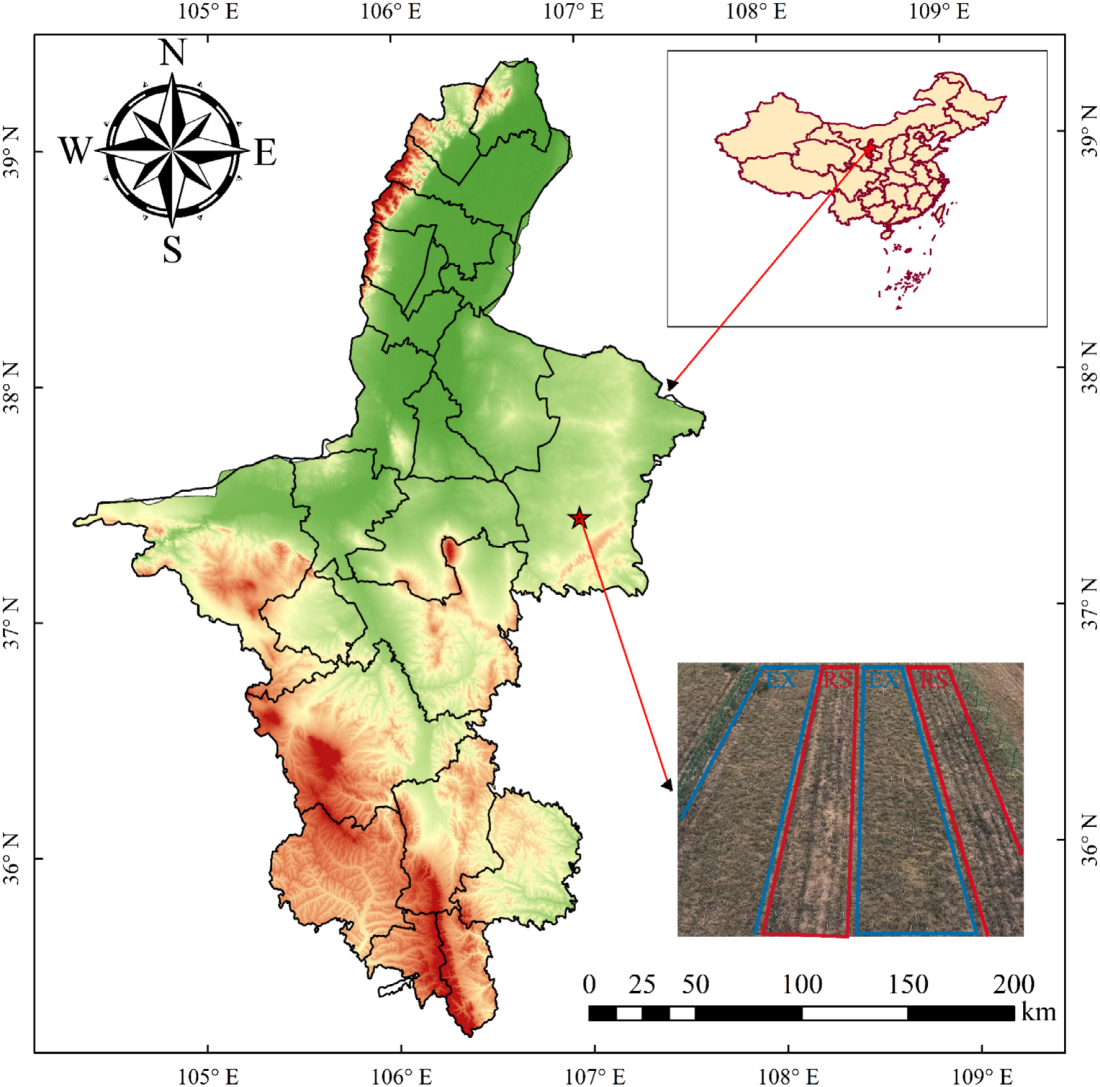
Overview and distribution of the sample plot of the study area. ★stands for experimental locations.

The study site is located in Dashuikeng Town, Yanchi County, Ningxia (37°28′–37°29′ N, 106°56′–106°57′ E, elevation 1523 m). The region is a transitional zone between the Mu Us Sandy Land and the Loess Plateau, with a typical temperate continental climate. The annual average temperature is 7.5°C, with precipitation concentrated from June to September, and August accounts for more than 40% of the total precipitation. The average annual precipitation over the past 60 years is about 298.3 mm, with the maximum reaching 582.8 mm and the minimum at 182 mm. The evaporation exceeds 2700 mm and the average frost‐free period is 164 days per year. The main soil types are light grey calcareous soil and aeolian sand, according to the soil classification method of the Natural Resources Conservation Service of the USDA (Figure 1) (Lu et al. 2023). Psammophytic vegetation and desert vegetation are the main types of vegetation, with dominant species such as *Agropyron mongolicum*, *Stipa breviflora* , *Lespedeza davurica* and *Artemisia scoparia* (Wu et al. 2020).

### 2.2 Experimental Design

Prior to 2002, the study area grasslands were used primarily for grazing, leading to desert steppe degradation due to overgrazing. In 2003, in order to protect natural desert steppe and restore degraded overgrazed areas, the government implemented year‐round grazing exclusion in the region. In 2016, the vegetation coverage measured 32.5% (Bardgett et al. 2021), indicating the test area is still in a state of degradation. So in May 2017, native species were reseeded based on the vegetation density of dominant plant species in nearby undegraded desert steppe. We collected the basic information of degraded grassland before restoration measures were implemented (0YEX(RS)) and established two types of restoration measures on degraded grasslands in the desert steppe: reseeded grassland (RS) and grazing exclusion grassland (EX). The ratio of Poaceae to Fabaceae plants was determined to be 6:4 based on vegetation survey data from nearby undegraded desert steppe. During the reseeding process on the desert steppe, distribute a seed mixture containing four native species at a rate of 2.5 kg ha^−1^. The seed mixture comprises 60% *Agropyron mongolicum*, 15% *Lespedeza potaninii* , 15% *Astragalus melilotoides* and 10% *Astragalus adsurgens* by weight. The seeding depth is 3 cm with a spacing of 20 cm.

### 2.3 Sampling and Measurements

We conducted vegetation collection in August 2020 and 2023, during the peak growth period of the plants. We set up 5 plots of 1 m × 1 m each for plant sampling and species identification in both the reseeding and grazing exclusion areas. We recorded the number of plant species, plant height, plant coverage, plant density, geographical location and elevation within each plot. The pin prick method was used to determine the relative projected area of each plant species, which was then used to calculate species coverage (Arjmandi et al. 2023; Sun, Wang, and Niu 2023; Gao et al. 2024; Mekhrovar et al. 2024; Tong et al. 2024; Wang et al. 2024). We calculated the average of ten random measurements of the natural height of each plant species within a plot to represent its height. We neatly cut off the aboveground parts of plants in the field, count them by plant species to calculate their density and pack them into envelopes to bring back to the laboratory. After placing them in a drying oven at 105°C for 30 min to kill the plants, we then dry them at 65°C for 48 h until a constant weight is reached, which represents the aboveground biomass (Wu et al. 2020; Sun et al. 2023).

### 2.4 Data Analysis

#### 2.4.1 Species Importance Values

The important value (IV) is a comprehensive quantitative indicator that measures the status and role of a species in a community (Zhang, Delgado‐Baquerizo, et al. 2023). A higher important value indicates a greater dominance of the species in the community. The relative important value is calculated using the species' relative abundance (RA), relative biomass (RB) and relative cover (RC).

$$\mathrm{IV}=\left(\mathrm{RA}+\mathrm{RB}+\mathrm{RC}\right)/3$$

#### 2.4.2 Diversity of Species

Based on the importance values, the following four indices are calculated to characterise the diversity of plant species in the desert steppe (Travlos et al. 2018): Margalef richness index (R), Shannon‐Weiner diversity index (H), Pielou evenness index (J) and Simpson dominance index (D) (Singh 2020; Negi et al. 2024). The specific formulas for calculation are as follows:

Margalef index:

$$R=\left(S-1\right)/\ln N$$

Simpson index:

$$D=\sum P_{i}^{2}$$

Shannon‐Wiener diversity index:

$$H=-\sum \left(P_{i}\times \ln P_{i}\right)$$

Pielou index:

J=H/lnS

where *S* is all the species numbers in the 1 m × 1 m quadrat, *N* is the total number of all species in each quadrat. *P*_*i*_ is the relative importance value of the species *i* based on the cover, abundance and biomass of the vegetation.

#### 2.4.3 Ecological Niche

(1) The width of the niche was calculated using the *Levins* index (regardless of resource status) (Chai et al. 2016; Yang et al. 2024).

$$B_{i}=\frac{1}{\sum_{j=1}^{s}{\left(P_{\mathrm{ij}}\right)}^{2}}$$

*B*_*i*_ is the breadth of the niche of species *i*, ranging from [0, ln*S*], *P*_*ij*_ is the proportion of the importance value of species *i* in resource *j* to the importance value of species *i* in all resources and *S* is the total number of resources (quadrats).

(2) Niche overlap was calculated by *Pianka's* index *O*_*ik*_ (Considering the difference of population niche breadth) (Hu et al. 2022; Juan et al. 2023).

$$O_{\mathrm{ik}}=\frac{\sum_{j=1}^{s}P_{\mathrm{ij}}P_{\mathrm{kj}}}{\sqrt{\left(\sum_{j=1}^{s}P_{\mathrm{ij}}^{2}\right)\left(\sum_{j=1}^{s}P_{\mathrm{kj}}^{2}\right)}}$$

*O*_*ik*_ is the overlap index of the niches of species *i* and k; *p*_*ij*_ and *P*_*kj*_ are the importance values of species *i* and *k* in the resource *j*, respectively.

#### 2.4.4 Overall Interspecific Association Analysis

The general association of the community was determined using the variance ratio method, and the statistic *W* was calculated to test whether the overall association was significant.

$$S_{T}^{2}=\frac{1}{N}\sum_{j=1}^{N}{\left(T_{j}-t\right)}^{2}$$

$$\delta_{T}^{2}=\sum_{i=1}^{S}P_{i}\left(1-P_{i}\right)$$

$$P_{i}=\frac{n_{i}}{N}$$

$$\mathrm{VR}=\frac{S_{T}^{2}}{\delta_{T}^{2}}$$

W=VR×N

*n*_*i*_ is the number of squares in which species *i* appears, and *N* is the total number of squares. *S* is the total number of species, *T*_*j*_ is the total number of species appearing in quadrat *j* and *t* is the average number of species in the quadrat. When the sample is independent and satisfies the null hypothesis, the *VR* expectation is 1. When *VR* = 1, the main species are not connected. When *VR* > 1, the main species are positively connected. When *VR* < 1, the main species are negatively connected. The statistic *W* was used to test the significance of VR deviation 1. When *χ* ^*2*^_*0.95*_ (*N*) < *W* < *χ* ^*2*^_*0.05*_ (*N*), the main species were not significantly correlated. Otherwise, the main species were significantly correlated.

#### 2.4.5 Interspecific Association Analysis

The overall correlation between plant species (*VR*) was calculated using the formula proposed by Schluter (1984) based on whether a species exhibits a particular characteristic in the sample plots. Dominance interactions between species were calculated using *χ* ^2^, *OI* index and *DI* index. The 2 × 2 contingency table was arranged according to the presence or absence of species pairs in the sample plots, where species 1 and species 2 are represented as $\left[\begin{array}{cc} a & b\\ c & d \end{array}\right]$ with *a* denoting the number of sample plots where both species are present, *b* and *c* representing the number of sample plots where only species 1 or species 2 is present and *d* representing the number of sample plots where neither species is present. To ensure consistency in the notation, it is typically required that *a* + *c* ≥ *a* + *b*; otherwise, the positions of the two species need to be swapped. Based on the values of *a*, *b*, *c* and *d*, the formula is as follows (Du et al. 2024):

##### 2.4.5.1 Chi‐Square Test

The interspecific association was calculated using the *χ* ^2^ chi‐square test, considering the discontinuity of sampling. The *χ* ^2^ value was determined using the Yates continuous correction method. See the formula below:

$$\chi^{2}=\frac{N{\left(\left|\mathrm{ad}-\mathrm{bc}\right|-0.5N\right)}^{2}}{\left(a+b\right)\left(c+d\right)\left(a+c\right)\left(b+d\right)}$$

*N* is the total number of squares. When *ad* > *bc*, there is a positive association between pairs of species, and when *ad* < *bc*, there is a negative association between pairs of species. If *χ* ^2^ < 1.15 (*p* > 0.05), it can be considered that the species pair is basically independent and the interspecific association is not significant. If 1.15 < *χ* ^2^ < 11.07 (0.01 < *p* < 0.05), it can be considered that there is a significant association between species pairs. If *χ* ^2^ > 11.07 (*p* < 0.01), it can be considered that there is a very significant association between species pairs.

##### 2.4.5.2 Association Coefficient

Percentage of Cooccurrence, Ochiai index and Dice index:

$$\mathrm{OI}=\frac{a}{\sqrt{\left(a+b\right)\left(a+c\right)}}$$

$$\mathrm{DI}=\frac{2a}{2a+b+c}$$

*OI* and *DI* indices can reflect the degree of positive association between species and can mitigate bias in the association coefficient *AC* caused by the influence of *d*, with values ranging from [0, 1]. When *a* = *S*, the coefficients are both 1, indicating the highest probability of species co‐occurrence in a sample plot. On the contrary, when *a* = 0, both indices are 0, signifying the absence of a positive association between species. The combined use of the *OI* and *DI* indices provides a more accurate assessment of the strength of positive associations between species. However, at low values of *a*, the presence of *d* is overlooked, amplifying the roles of *a*, *b* and *c*, thus hindering the assessment of the strength of negative associations between species.

##### 2.4.5.3 Pearson Correlation

Pearson's coefficient provides an accurate and objective measure of the linear relationship between species using quantitative data. The calculation of the Pearson correlation coefficient is based on the following formula (Juan et al. 2023):

rsi,k=[∑j=1NXij−xi¯Xkj−xk¯∑j=1NXij−xi¯2Xkj−xk¯2

*r*_*s(i, k)*_ is the Pearson correlation coefficient between species *i* and species *k*, *N* is the total number of quadrats, and *X*_*ij*_ and *X*_*kj*_ are the abundances of species *i* and species *k*, respectively. They form vectors *x*_*i*_ and *xk* and are the average values of the important values of species *i* and *k* in the *j*‐cube, respectively. The value of *r*_*s(i, k)*_ is positively correlated in (0, 1], the value of *r*_*s (i, k)*_ is negatively correlated in [−1,0) and the value of *r*_*s(i,k)*_ is not correlated when it is 0.

#### 2.4.6 Community Biomass Stability

The biomass of a community is a key indicator of the succession and stability of community development (Tilman 1999). In ecology, the term for the temporal stability of community biomass varies, such as temporal stability and biomass stability, but essentially corresponds to the coefficient of variation in biostatistics. This metric is used to compare the variability of observed values within the same group across different units and scales (Basto et al. 2015; Chen et al. 2023; Evans et al. 2023). This study extends the calculation method of this indicator to the assessment of the stability of the temporal biomass of the community, aiming to investigate its relationship with diversity and interspecies interactions within the community. To avoid ambiguity, the term ‘community stability’ will be used uniformly in the following text. Stability of plant communities is indicated by the reciprocal of the coefficient of variation (CV) of species biomass within the community (ICV):

T=μ/σ

*μ* is the inter‐annual mean biomass of all species in the plot, and *σ* is the inter‐annual standard deviation of the biomass of each species in the corresponding plot.

### 2.5 Statistical Analysis

Statistical analysis was performed in Microsoft Excel 2022 and R 4.3.2. In Microsoft Excel 2022, we organised and summarised the plot data, calculated species diversity indices and plotted graphs using Origin Pro 2024. In R Studio 4.3.2, the ‘spaa’ package was utilised to compute species association indices and niche analysis, while the ‘ggplot2’ and ‘corrplot’ packages were used to visualise the related data.

Partial least squares path modelling (PLS‐PM) was used to assess the direct and indirect relationships among community species diversity, niche width, niche overlap, interspecific linkage coefficient and community stability. This model does not rely on the normal distribution of the data, can be implemented with limited data and can compute the paths between latent variables and their complex causal relationships. Structural equation path analysis is a multivariate method used to examine and measure the various connections and influences among groups of variables, thereby examining the impact of interrelated variables on explanatory variables to avoid unnecessary path confusion. Path coefficients indicate the direction and strength of the linear relationship between latent variables and explained variance (*R* ^2^), calculated through the model. Goodness of fit (GOF) statistics are used to evaluate the overall predictive capacity of the model, with a GOF value > 0.7 considered acceptable for PLS‐PM. We constructed a model using Partial Least Squares Path Modelling (PLS‐PM) with Smart PLS 3.0.

## 3 Results

### 3.1 Composition and Diversity of Species in the Plant Community

After restoration of degraded desert steppe through reseeding (RS) and grazing exclusion (EX), plant community species tend to become more similar as the community progresses through succession. A total of 30 plant species were monitored in the desert steppe through years of cumulative sampling (Table S1). Gramineae and legumes dominated the community composition (Table S2).

With the increase in restoration years, the proportion of important value of RS gramineous plants increased greatly, while the proportion of leguminous plants decreased and gradually approached EX, but the proportion of EX forbs increased. The results showed that with the succession of the reseeding community in the desert steppe, the dominant species gradually changed from the legume species *L. potaninii* to the gramineous species *A. mongolicum* . *A. mongolicum* is the dominant species in the grazing‐forbidden desert steppe community and can undergo stable succession.

**FIGURE 2:**
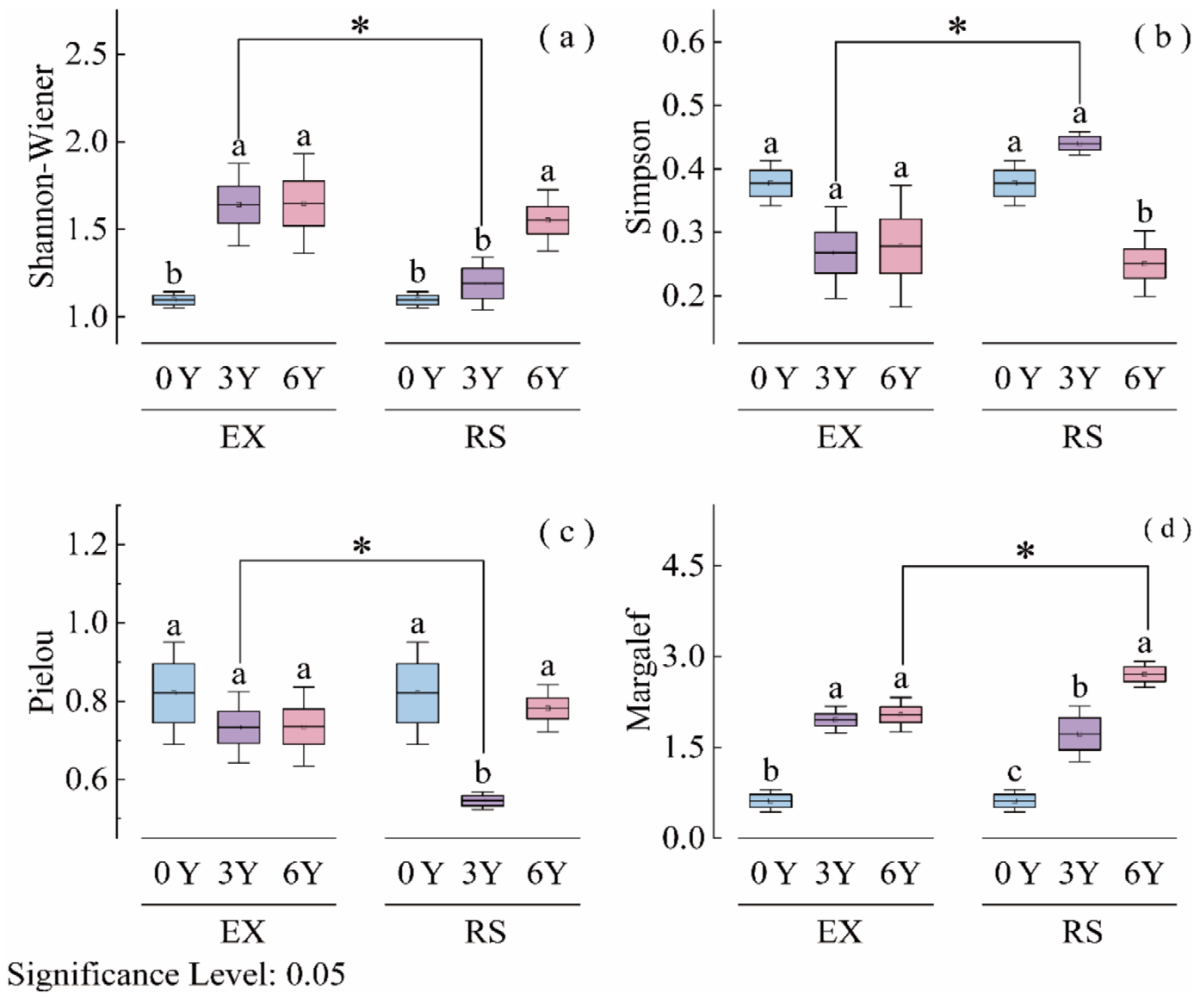
Effects of grassland reseeding on Shannon‐Wiener diversity index, Simpson index, Pielou index and Margalef index of community species. 0Y, Degradation grassland; 3Y, Treatment for degradation repair for 3 years; 6Y, Degradation repair treatment for 6 years; EX, Grazing exclusion grassland; RS, Reseeded grassland. Boxplots with the different letters were significantly different in LSD multiple range tests reported from one‐way ANOVA (*p* < 0.05). An asterisk represents a significant difference (*p* < 0.05) between the reseeded and grazing exclusion treatments according to Student's *t* test.

The species diversity of the RS and EX plant communities has changed significantly compared to desert steppe of 0YEX (RS) (*p* < 0.05) (Figure 2). According to our study, the Shannon‐Wiener index of native species in reseeded desert steppe has been consistently rising over time (*p* < 0.05) (Figure 2a). Likewise, the diversity of species tends to grow annually as the reseeding years progress (*p* < 0.05) (Figure 2d).

### 3.2 Niche Width and Overlap of Plant Species

#### 3.2.1 Niche Width

In each quadrat, we selected plant species with an importance value > 1% to analyse changes in community species' niche and interspecies relationships.

Our results show that the change in niche width of RS grasses increased with the increase in resowing years, while the opposite was observed for legumes. The niche breadth of EX leguminous plants increased with the increase in the years, whereas the change in niche breadth of gramineous plants was opposite. Niche breadth of RS species *A. mongolicum* gradually increased during community succession, and the niche breadth of *L. potaninii* decreased, while that of *A. laxmannii* gradually decreased until it disappeared during succession. Correlation tests between niche breadth and species importance show that RS, EX and 0YEX (RS) are significantly positively correlated with niche breadth and species importance in grasslands (*p* < 0.05) (Tables S2 and S3, Figure S1).

#### 3.2.2 Niche Overlap

With the increase of restoration years, RS niche overlap gradually decreased, and EX basically did not change.

**FIGURE 3:**
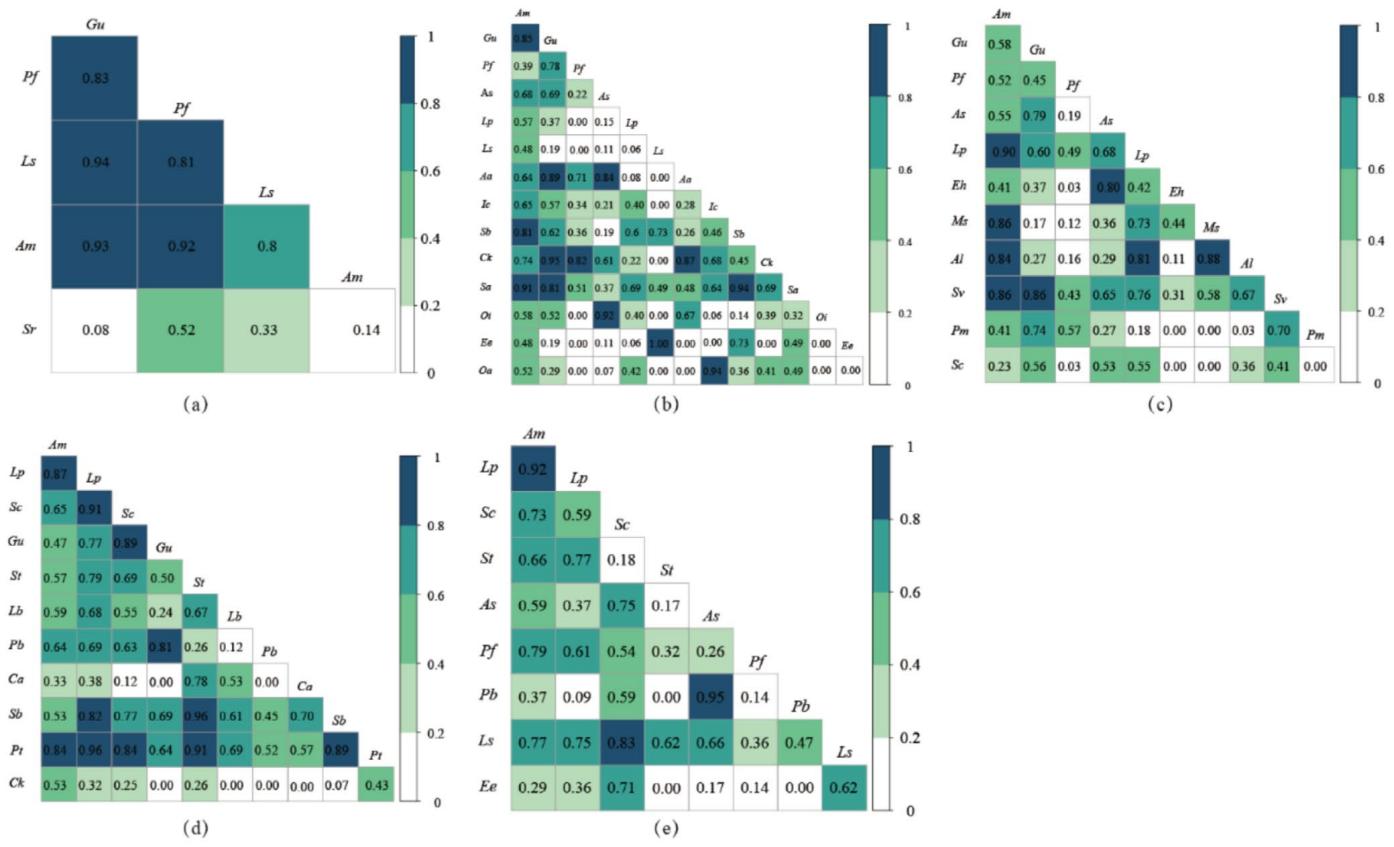
Overall interspecific association of the reseeding grassland community. The closer the colour is to green, the closer the overlap value is to 1 and the closer the colour is to white, the closer the overlap value is to 0. (a) Degradation grassland (0YEX(RS)); (b) After 3 years of grassland grazing exclusion (3YEX); (c) After 3 years of grassland reseeding (3YRS); (d) After 6 years of grassland grazing exclusion (6YEX); (e) After 6 years of grassland reseeding (6YRS).

The ecological niche overlap results indicate that the average overlap for 3YRS is 0.45. Specifically, 41 pairs have an overlap value below 0.6, making up 75.93% of the total pairs, while 8 pairs have an overlap value above 0.8, representing 14.82% of the total pairs (Figure 3). The primary species overlap consists of *A. mongolicum* and *L. potaninii* (0.90), followed by *A. laxmannii* and *M. sativa* (0.88), with *L. potaninii* being the dominant species. The average niche overlap of 3YEX is 0.41, with 61 pairs having overlap values < 0.6, accounting for 67.78% of the total pairs. Additionally, there are 12 pairs with overlap values > 0.8, accounting for 13.00% of the total pairs. The primary species overlap consists of *L. secalinus* and *E. esula* (1.00), *G. uralensis* and *C. komarovii* (0.95), followed by *S. bungeana* and *C. serotina* (0.94), *O. aciphylla*a and *I. chinensis* (0.94). The average niche overlap of 6YRS is 0.48, with 21 pairs having overlap values < 0.6, accounting for 58.33% of the total logarithm and 3 pairs having overlap values > 0.8, accounting for 8.33% of the total logarithm. The primary species overlap consists of *A. scoparia* and *P. bifurca* (0.95), *A. mongolicum* and *L. potaninii* (0.92), *L. secalinus* and *S. collina* (0.83), with *A. mongolicum* being the dominant species. The average niche overlap of 6YEX is 0.53, with 28 pairs having overlap values < 0.6, accounting for 50.90% of the total logarithm, and 11 pair shaving overlap values > 0.8, accounting for 20.00% of the total logarithm. The primary species overlap consists of *S. breviflora* and *S. bungeana* (0.96), *L. potaninii* and *P. tenuifolia* (0.96). The average 0YEX(RS) niche overlap is 0.63, with 4 pairs having overlap values < 0.6, accounting for 40.00% of the total logarithm. The primary species overlap consists of *L. secalinus* and *G. uralensis* (0.94).

In general, there are variations in the ecological niche overlap among different plant species. RS niche overlap is primarily driven by competition among dominant species. As the number of years of reseeding increases, the logarithmic ratio of high niche overlap values decreases, resulting in a gradual stabilisation of overlap levels. EX species resource competition level has become higher. As the years of grazing exclusion have increased, the niche overlap has gradually increased.

### 3.3 Interspecific Association of Plant Species

#### 3.3.1 Overall Connectivity of Community Species

The overall variance ratios for the main species in the community are presented in table (Table 1). EX the variance ratios are all < 1, while for RS they are all > 1. The variance ratio is equal to 1 for 0YEX(RS), indicating a positive association for the RS plant communities, a negative association for EX and no association for 0YEX(RS). The test statistic W, which measures the deviation of the VR value from 1, indicates that there is a positive correlation among species in the RS community and a negative correlation among species in the EX community.

**TABLE 1:** Effect of reseeding on the overall interspecific association of the community.

Treatment	*S*	Variance ratio	Statistic *W*	*χ* ^2^_(0.05, 0.95)_	Community relevance results
0YEX(RS)	5	1	3	(1.15, 11.07)	No association
3YEX	14	0.65	3.27	(1.15, 11.07)	Negative connection
3YRS	11	1.59	7.94	(1.15, 11.07)	Positive association
6YEX	11	0.53	2.67	(1.15, 11.07)	Negative connection
6YRS	9	1.82	9.09	(1.15, 11.07)	Positive association

Abbreviations: 0YEX(RS), Degradation grassland; 3YEX, After 3 years of grassland grazing exclusion; 3YRS, After 3 years of grassland reseeding; 6YEX, After 6 years of grassland grazing exclusion; 6YRS, After 6 years of grassland reseeding.

#### 3.3.2 Pair Associations of Plant Community Species

**FIGURE 4:**
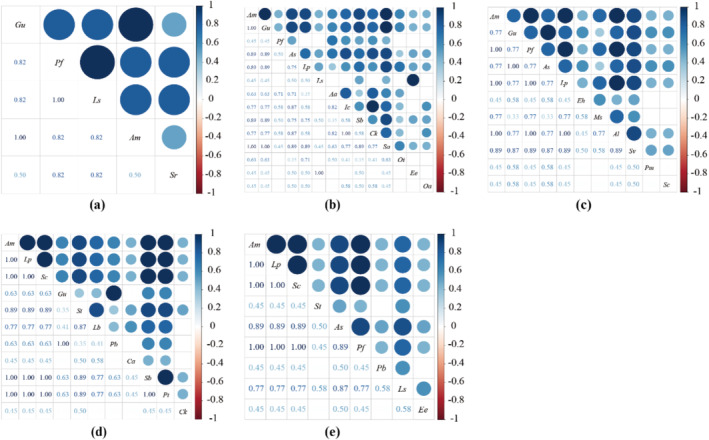
Effects of different reseeding years on the ochiai index (*OI*) value of the community. (a) Degradation grassland (0YEX(RS)); (b) After 3 years of grassland grazing exclusion (3YEX); (c) After 3 years of grassland reseeding (3YRS); (d) After 6 years of grassland grazing exclusion (6YEX); (e) After 6 years of grassland reseeding (6YRS).

**FIGURE 5:**
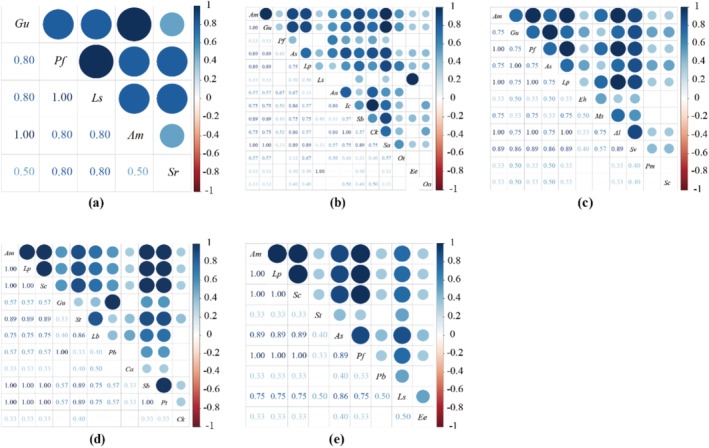
Effects of different reseeding years on the dice index (*DI*) value of the community. (a) Degradation grassland (0YEX(RS)); (b) After 3 years of grassland grazing exclusion (3YEX); (c) After 3 years of grassland reseeding (3YRS); (d) After 6 years of grassland grazing exclusion (6YEX); (e) After 6 years of grassland reseeding (6YRS).

**FIGURE 6:**
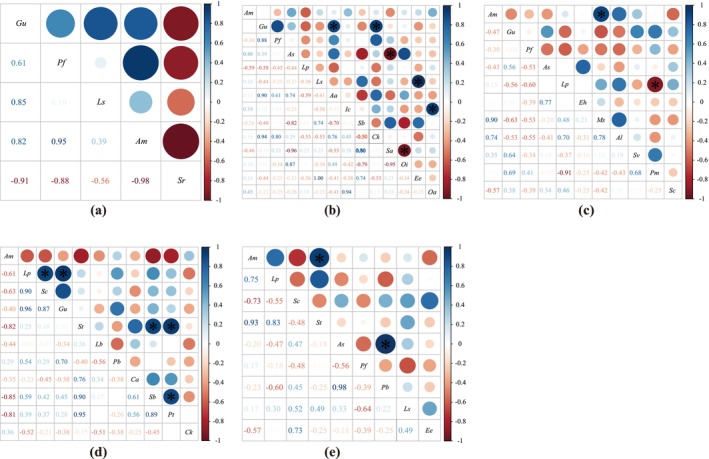
Effects of different reseeded years on Pearson's correlation coefficient and significance. (a): Degradation grassland; (b): Investigation of enclosed grassland in 2020; (c): Investigation of reseeded grassland in 2020; (d): Investigation of enclosed grassland in 2023; (e): Investigation of reseeded grassland in 2023.

We used OI and DI indices to distinguish the association strength of species pairs after reseeding on desert steppe (Figures 4 and 5). We found that in each treatment, there are 6 pairs (0YEX(RS)), 13 pairs (3YEX), 6 pairs (3YRS), 6 pairs (6YEX) and 5 pairs (6YRS) with both OI and DI values > 0.8, accounting for 60.00%, 14.28%, 10.91%, 10.91% and 13.89%, respectively. The results of the Pearson's correlation coefficient test indicate a significant change in the interspecific correlation of RS as the repair years increase (Figure 6).

There were 31 pairs of negatively correlated logarithms and 23 pairs of positively correlated logarithms in 3YRS, accounting for 56.36% and 41.82% of the total logarithms, respectively. Significant associations are observed in 2 pairs of species. *L. potaninii* and *P. miliaceum* shows significant negative correlations, while *A. mongolicum* and *M. sativa* show significant positive correlations, accounting for 1.81% of total logarithms (*p* < 0.05) (Figure 6c). There are 51 pairs of negatively correlated logs and 37 pairs of positively correlated logs in 3YEX on interspecific association, representing 56.04% and 40.66% of the total logs, respectively. There are 6 species pairs with a significant degree of association. *A. scoparia* and *S. bungeana*, and *O. leucophylla* and *S. bungeana* showed significant negative associations; *A. altaicus* and *G. uralensis* , *C. komarovii* and *G. uralensis* , *L. secalinus* and *E. esula* , and *I. chinensis* and *O. aciphylla* showed a significant positive association, accounting for 2.20% and 4.40% of the total logarithm, respectively (*p* < 0.05) (Figure 6b).

There are 21 pairs of negatively correlated logs and 15 pairs of positively correlated logs in 6 years on interspecific association, representing 58.33% and 41.67% of the total logs, respectively. *A. mongolicum* and *S. breviflora* , *A. scoparia* and *P. bifurca* showed a significant positive association, 5.56% of the total logarithm (*p* < 0.05) (Figure 6e). There are 27 pairs of negatively correlated logs (49.09%) and 26 pairs of positively correlated logs (47.27%) in 6YEX on interspecific association, representing 49.09% and 47.27% of the total logs, respectively. *L. potaninii* and *S. collina* , *L. potaninii* and *G. uralensis* , *S. breviflora* and *C. serotina* , *S. breviflora* and *P. tenuifolia* , *C. serotina* and *P. tenuifolia* showed significant positive association, 9.09% of the total logarithm (*p* < 0.05) (Figure 6d). There are 4 pairs of negatively correlated logarithms and 6 pairs of positively correlated logarithms in 0YEX(RS) on interspecific association, accounting for 44.00% and 60.00% of the total logarithm, respectively (Figure 6a).

The results of the Pearson's correlation coefficient test are basically consistent with the high logarithmic overlapping ratio of the OI and the DI index, and RS has a significant correlation with only a few species compared to EX, indicating that EX has a high demand for environmental resources and a strong competitive relationship (Figures 4, 5, 6).

### 3.4 Factors Influencing Community Stability

**FIGURE 7:**
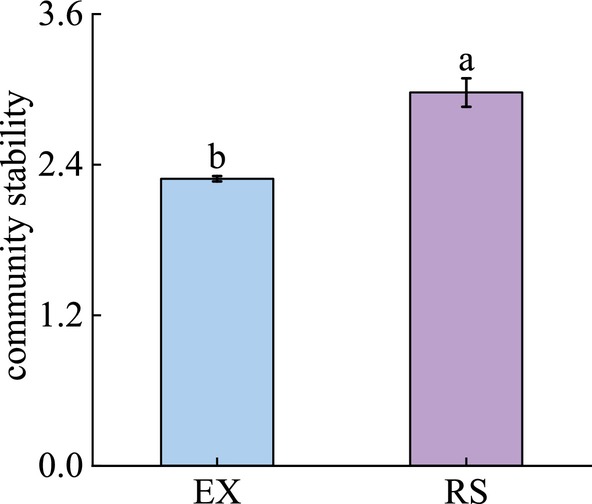
Effects of reseeded on the stability of desert steppe communities. EX, Grazing exclusion grassland; RS, Reseeded grassland. The same lowercase letters mean insignificant differences between treatments (*p* > 0.05).

**FIGURE 8:**
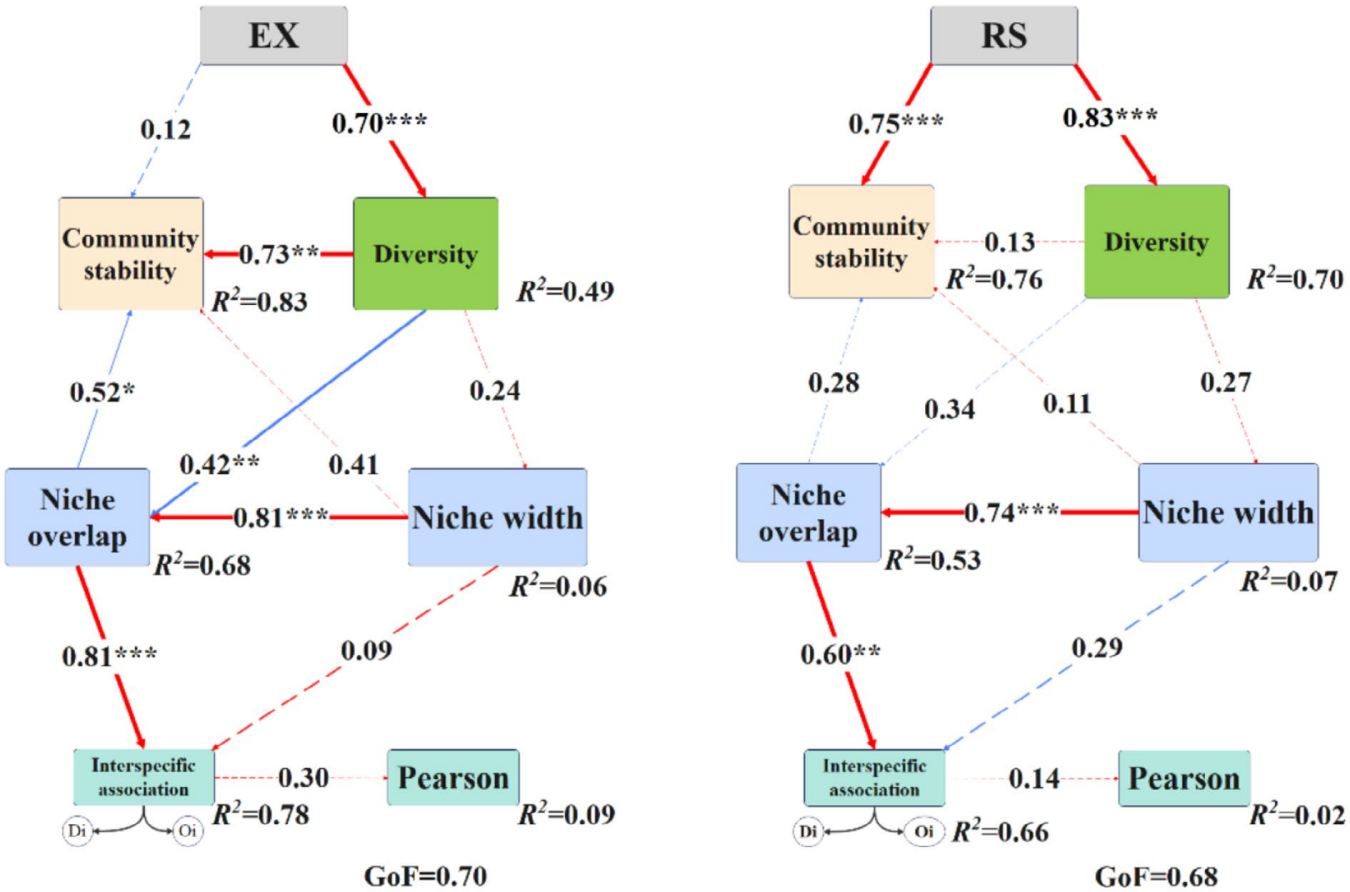
Partial least squares path model (PLS‐PM) showed the effects of species diversity, niche and interspecific association on community stability. Red lines represent significant positive affect paths; blue lines represent a significant negative affect path (*p* < 0.05); grey dashed lines represent a non‐significant affect path (*p* > 0.05). The width of the arrows is proportional to the strength of the path coefficients, and the adjacent numbers on the arrows indicate the standardised path coefficients. *R* ^2^ represents the determination coefficient of each variable in the inner model. ***p < 0.001; **p < 0.01; *p < 0.05. Goodness‐of‐fit (GOF) index indicates the overall prediction performance.

The results of community stability changes after reseeding of native grass species are shown in the figure (Figure 7). Community stability of RS was significantly higher than that of EX. PLS‐PM is part of the path analysis. Path analysis is a multivariate method that is used to examine and measure a variety of connections and effects between groups of variables. We used the partial least squares path model analysis method (PLS‐PM) to model the relationship between species diversity, niche, interspecific association and community stability of grassland communities. The complexity of the interactions between variables was reduced by the partial least squares path model (PLS‐PM) to more clearly identify the direct and indirect effects of the diversity, niche and interspecific association of the community species on community stability (Figures 7 and 8).

Utilising both the Shannon‐Wiener index and the Margalef index for PCA clustering resulted in a single diversity index (Table S4). The GOF coefficients of PLS‐PM for EX and RS showed that the prediction results of the model were better. The model fitting coefficients were 0.70 and 0.68, respectively, and the *R* ^2^ of community stability was 0.83 and 0.76, respectively, indicating that the model was able to explain 83% and 76% of the variance in community stability. According to the outcomes of the PLS‐PM model, both EX and RS exhibited direct and significant positive effects on diversity (*p* < 0.05). Similarly, niche breadth had a direct and significant positive effect on niche overlap, which in turn had a direct and significant positive effect on the interspecific association coefficient (*p* < 0.05). While EX did not show a significant positive impact on community stability, diversity was found to have a direct and significant positive effect on community stability and a direct and significant negative effect on niche overlap (*p* < 0.05).

The investigation of the total effects (direct and indirect) shows that the EX model primarily has an indirect impact on community stability (Direct effect: −0.12, Indirect effect: 0.66), while it has a direct effect on diversity (Direct effect: 0.70). Diversity has a direct effect on community stability (Direct effect: 0.73), and niche width has a direct effect on community stability (Direct effect: 0.41) and on niche overlap (Direct effect: 0.81). Additionally, niche overlap has a direct effect on community stability (Direct effect: −0.52). On the other hand, the RS model has a predominant direct impact on community stability (Direct effect: 0.65, Indirect effect: 0.17) and has a direct effect on diversity (Direct effect: 0.83) (Tables S5 and S6).

## 4 Discussion

### 4.1 Important Value and Diversity of Species

Species importance values are often used to reflect the dominance of individual plants in the community, and these values can impact species diversity within the community (Granot and Belmaker 2020; Juan et al. 2023). Species diversity is often used as an important indicator of the restoration of degraded ecosystems (Hai et al. 2020). We used to restore degraded grasslands through various measures such as reseeding, fertilisation, irrigation and exclusion (Zhang et al. 2019). Research has found that both reseeding and exclusion can promote the ecological restoration of degraded desert steppe. Among these methods, reseeding has become increasingly popular in the northern China desert steppe for restoring vegetation (Yue, Shi, et al. 2021; Zhang, Fan, et al. 2023; An and Li 2024). The key to successful grassland restoration through reseeding is selecting the appropriate species (Zhang, Zhu, et al. 2020). In this study, the reseeded species mainly consist of native grass species such as *S. collina* , *L. potaninii* , *A. mongolicum* and *S. breviflora* . Among them, *L. potaninii* is a semi‐shrub of the perennial leguminous Lespedeza, and *A. mongolicum* is a perennial grass. *A. mongolicum* belongs to the genus Agropyron of the Gramineae family. Both of these species have strong drought tolerance and are excellent forages for vegetation restoration in the desert steppe (Xu et al. 2019; Yu, Guo, and Sun 2021; Gao et al. 2022). From the reseeded grassland community, it can be inferred that the species importance value of the Poaceae species *A. mongolicum* and the Fabaceae species *L. potaninii* are strong competitors for resources. Additionally, *L. potaninii* is able to maintain stability during community succession.

Our research results show that both reseeded and grazing exclusion desert steppes significantly increased species diversity, with Margalef index EX and RS significantly higher than those of the control (0YEX(RS)) desert steppe, consistent with previous studies (Zhan et al. 2022; Mi et al. 2024). This may be due to the introduction of reseeded species, which disrupted the survival environment of some original species, breaking the original structure and spatial pattern of the local vegetation and creating a new ecological niche pattern (Jiang et al. 2024). The reseeded native species were also dominant species in the original plant community, resulting in a few species gaining a larger ecological niche (Mi et al. 2024). In the initial stages of desert steppe reseeded, species are adapting to the environment and resources are directed towards the reseeded species. This may result in a slow increase in species diversity, and the diversity and richness of species in the early stages of reseeding may be lower than in the original ecosystem. Over time, plant communities recover their succession and gradually increase in diversity and richness, in line with previous studies (Sun et al. 2020; Zhang et al. 2022).

Reseeding increases external seed sources, leading to competition for limited soil resources and inhibiting the growth of weed seeds, thus reducing the overall richness of the community (Basto et al. 2015). At the same time, the reintroduction of native grass species has improved the problem of low seed yields of existing plants, allowing them to occupy a more prominent position in the community and regain a competitive advantage (Buchmann et al. 2018). Consequently, reseeding desert steppe exhibited a noticeable change in species richness and diversity over time compared to enclosed desert steppe, ultimately improving the rate of vegetation restoration in desert steppe.

### 4.2 Effects of Reseeding on Species' Niche

Species importance value (*IV*) is closely tied to its ecological niche width (Juan et al. 2023; Du et al. 2024), the ecological niche width is a measure of a species' ability to access resources and adapt to its environment within the community (Pshegusov et al. 2021; Chen et al. 2024). Using the importance value and ecological niche width, we can evaluate the mechanisms behind the loss of grassland species (Band et al. 2022). Theoretically, the wider the ecological niche width, the more evenly distributed the species, and the stronger the adaptability and resource acquisition ability (Zhang, Dong, et al. 2020; Zhang, Xu, et al. 2020). Ecological niches overlap when multiple species share or compete for available resources in the same time and space. Ecological niche overlap and width are both factors that determine the co‐development of species (Costa‐Pereira et al. 2019; Juan et al. 2023). In this study, the species with the widest niche width in terms of RS changed from *L. potaninii* (4.55) to *A. mongolicum* (4.61) as the years progressed. Similarly, the species with the widest niche width in terms of EX changed from *A. mongolicum* (4.82) to *L. potaninii* (4.70) with increasing years. Species with higher importance values also tend to have larger niche widths. In our study, there was a significant positive correlation between importance values and niche width. However, *A. mongolicum* (0.45), with the highest species importance value in 6YEX, did not have the widest niche breadth, while *L. potaninii* (0.13) had a wide niche breadth. This difference may be due to the distribution of resources among species and the influence of environmental factors on niche breadth. Niche width is determined by both the species itself and environmental factors (Gu, Gong, and Li 2017; Juan et al. 2023). Species with higher importance values may obtain larger niche widths, but not necessarily always (Cardoso, Ladio, and Lozada 2017; Wagner et al. 2017). The importance value of species in the desert steppe is closely related to their ecological niche width. Reseeded with native grass species can alter the soil seed environment, thus affecting the species' importance value and ecological niche. This can enhance the competitive ability of superior seeds for resource utilisation, which is crucial for explaining the protection and improvement of desert steppe from an ecological niche perspective.

The lower the level of niche overlap, the weaker the interaction ability between species, allowing species to stably coexist. The study area is in a semi‐arid climate, where the dry environment reduces the level of niche overlap, and herbaceous plants typically have lower niche levels (Guo et al. 2018; Xu et al. 2021). In this study, RS shows that the fraction of high niche overlap gradually decreases as the number of years of coexistence increases. EX ecological niche overlap ratios did not show a downward trend. The height ecological niche overlap suggests that the two species have similar life strategies and ecological requirements for resources and environments (Fielding and Pantel 2020). High ecological overlap inevitably leads to intense competition among species. Through adaptation to different ecological changes and evolutionary pressures, species form feedback regulation mechanisms that drive community development and succession (Tsafack et al. 2021). The climate in the desert steppe is dry, resulting in a high competition for resources between species. Natural recovery through grazing prohibition and enclosure takes a long time (Gu et al. 2022), and the restoration effect is easily affected by environmental changes (Li et al. 2013). Reseeding native grass species in desert steppe can help alleviate the intensity of interspecies resource competition and reduce the level of ecological niche overlap compared to grazing exclusion steppe. This is particularly evident in the changes of *L. potaninii* , *A. mongolicum* and *S. breviflora* . By promoting the coordinated use of resources among species and reducing competition levels, community succession gradually becomes more stable.

### 4.3 Effects of Reseeding on Interspecific Association

The overall connectivity of a community can reflect the stability of its structure. A low level of interconnection between species indicates an unstable community structure. As succession progresses, species compete and cooperate to maximise resource utilisation, leading to gradual stabilisation of species composition and a more stable community structure. Eventually, development leads to a top‐level community (Ma et al. 2019; Juan et al. 2023). Species coexisting in a plant community typically exhibit positive relationships, suggesting that they share similar environmental resource requirements, have strong complementarity, and utilise resources in a rational and efficient manner (Yuan et al. 2018). Conversely, when species show a preference for different environmental conditions and acquire resources heterogeneously, the overall community connection becomes negative, leading to niche separation (Ma et al. 2019; Pastore et al. 2021).

We used the variance ratio‐based OI index, DI index and Pearson's correlation coefficient to reveal how interspecific associations in the desert steppe ecosystem change after reseeding. The overall connectivity, EX showed negative associations, while RS showed positive associations. 0YEX(RS) steppe did not show connectivity associations, indicating intense competition among species in EX. This could lead to an incomplete and dynamically unbalanced community structure over time (Du et al. 2024). However, RS can break this imbalanced state by introducing some superior species, redistributing resources among plant species and promoting the allocation of limited resources to dominant species. This can result in increased resource availability for dominant species, but in the short term, it may lead to the elimination of weaker species in the reseeded grassland community, causing a decrease in species diversity and richness. The results of the interspecific association analysis in this study align with the findings of the χ2 test. In the reseeded grassland community, the majority of associations are positive, with only a few species exhibiting significant negative associations during the early stages of reseeding. The research suggests that the occurrence of negative associations is intricate and often linked to species' ecological niches, species pair distribution frequencies and habitat relationships (Gu, Gong, and Li 2017; Shao and Zhang 2021). With the increase in reseeding years, the number of negative association pairs gradually decreased, and significant negative association pairs disappeared. The proportion of positive association pairs increased, accompanied by a decrease in the proportion of high niche overlap. Although the negative association pairs in the grazing exclusion steppe gradually disappeared, the niche overlap still remained high. This shows that as reseeding and grazing exclusion take place, the strength of interspecies competition changes over time during community succession, but this cannot be achieved in a short period of time. Furthermore, the reseeding of indigenous grass species can mitigate the level of competition between plants, thus promoting the stability of grassland communities.

Competition may be unfriendly to individual species, but it can neutralise interspecific relationships and promote stability and adaptation of the community as a whole (Stubbs and Bastow Wilson 2004; Nathan 2006). Therefore, the desert steppe can be restored by supplementing with native grass species, increasing the efficiency of resource utilisation by adding high quality native plants and promoting the stable development of plant communities.

### 4.4 Factors Influencing Community Stability

Extensive research has demonstrated the ability of reseeding to mitigate steppe degradation and aid in its restoration (Wang et al. 2016; Qi et al. 2024). However, there is a lack of studies on the impact of the reseeding of native grass species on the structure and stability of the community. Changes in community stability manifest themselves through alterations in the average biomass and standard deviation, as different species in the community regulate stability through competition and symbiosis. Studies indicate that the stability of the plant community is primarily influenced by interspecific competition, environmental stress and disturbance activities (Lisner et al. 2024; Xu et al. 2024). Human intervention usually impacts species composition, which in turn changes the competitive interactions and interspecific relationships within the community. Research has shown that changes in interspecific effects significantly influence community stability, with beneficial interspecific effects that promote community stability (Kawatsu and Kondoh 2018). Also, reseeding species can obtain higher biomass, and, as they behaved according to Taylor's power law, they became more stable. Higher stability of reseeding species is also connected with a pure statistical effect where a synergistic increase in biomass and number of independent individuals results in the average of their stochastic variability and stabilisation of the whole population (Segrestin and Leps 2022; Lisner et al. 2024). Altogether, reseeding native grass seeds in steppe can accelerate the formation of stable grassland communities compared to grazing‐excluded steppe.

Throughout this investigation, it was observed that the stability of the grassland community in the reseeding steppe was significantly higher than that of the grazing exclusion steppe. Moreover, the Partial Least Squares Path Modelling (PLS‐PM). The combined effect of niche and diversity indices was identified as the primary driver in altering community stability (Evans et al. 2023). After restoration of vegetation, with an increase in years, the demand for natural environmental resources such as light, water and nutrients increases. However, limitations in resources can lead to increased competition (both within and between species). The restoration process is accompanied by community succession, where plants with stronger competitive abilities for resource acquisition intensify competition, leading to a decrease in the ecological niche scale of other species, thereby affecting community stability (Deng, Zhang, and Shangguan 2014; Chen et al. 2023). Reseeding native grass species in the desert steppe can enhance the utilisation of resources. Through resource complementarity, interspecific competition can be reduced, promoting the coexistence of species (Wu et al. 2022). Strengthening the coexistence of neighbouring species with dominant species can achieve neutral competition in the community, promoting healthy community development (Levine et al. 2017; Usinowicz et al. 2017; Majer et al. 2024).

## 5 Conclusions

Reseeding native grass species in degraded steppe can enhance the importance value and diversity index of species in desert steppe compared to grazing exclusion steppe. Over time, it can increase the ecological niche breadth of community species and reduce the level of ecological niche overlap in arid desert steppe. Analysis of interspecific association showed that the reseeding of native grass species in the desert steppe was in a relatively stable development and that the stability of the community increased steadily with time. Combined with the PLS‐PM analysis, it is proven that community stability is affected by the niche, the interspecific association index and the diversity of species, which can provide guidance for the restoration of degraded desert steppes in arid areas.

## Author Contributions

**Haixin Jiang:** conceptualization (lead), data curation (lead), investigation (lead), methodology (lead), software (lead), validation (lead), visualization (lead), writing – original draft (lead). **Yao Zhou:** funding acquisition (equal), methodology (equal), resources (supporting). **Wen Li:** investigation (equal), software (equal), visualization (equal). **Qi Lu:** investigation (supporting). **Dongmei Xu:** data curation (supporting), investigation (supporting). **Hongbin Ma:** data curation (lead), funding acquisition (supporting), methodology (lead), project administration (lead), supervision (lead), writing – review and editing (lead). **Xingzhong Ma:** funding acquisition (supporting), investigation (supporting). **Xiaowu Tian:** funding acquisition (supporting), investigation (supporting).

## Conflicts of Interest

The authors declare no conflicts of interest.

## Supporting information


**Figure S1** Analysis of niche breadth and species importance value of reseeding grassland. (a) Degradation grassland (0YEX(RS)); (b) After 3 years of grassland grazing exclusion (3YEX); (c) After 3 years of grassland reseeding (3YRS); (d) After 6 years of grassland grazing exclusion (6YEX); (e) After 6 years of grassland reseeding (6YRS). The same as below.
**Table S1** Composition of species and the life form of the plant community.
**Table S2** Species importance value (*IV*) of reseeding grassland species in desert steppe. ‘—’ Represents no species present.
**Table S3** Levins niche widths of reseeding grassland species in the desert steppe. ‘—’ Represents no species present.
**Table S4** Loads of Shannon‐Weiner index and Margalef index on the first two principal components of principal component analysis (PCA). The first principal component is PC1; PC2, the second principal component.
**Table S5** Direct, indirect and overall impacts of grazing exclusion on community stability.
**Table S6** Direct, indirect and total impacts of reseeded on community stability.

## Data Availability

The authors confirms that the data supporting the results of this study can be obtained in the Supporting Information of the article. The original data and R code used in this study can be obtained in FigShare: https://figshare.com/s/6b7cf1323e5f98995630.
