# Supplementary material for: Reseeding Native Species Promotes Community Stability by Improving Species Diversity, Niche, and Interspecific Relationships in the Desert Steppe of Northwest China
Source: Ecol Evol. 2025 Feb 5;15(2):e70929. doi: 10.1002/ece3.70929 (PMC11799735; doi:10.1002/ece3.70929)
Supplement: Supplementary file 1 — Figure S1 Analysis of niche breadth and species importance value of reseeding grassland. (a) Degradation grassland (0YEX(RS)); (b) After 3 years of grassland grazing exclusion (3YEX); (c) After 3 years of grassland reseeding (3YRS); (d) After 6 years of grassland grazing exclusion (6YEX); (e) After 6 years of grassland reseeding (6YRS). The same as below. Table S1 Composition of species and the life form of the plant community. Table S2 Species importance value (IV) of reseeding grassland species in desert steppe. ‘—’ Represents no species present. Table S3 Levins niche widths of reseeding grassland species in the desert steppe. ‘—’ Represents no species present. Table S4 Loads of Shannon‐Weiner index and Margalef index on the first two principal components of principal component analysis (PCA). The first principal component is PC1; PC2, the second principal component. Table S5 Direct, indirect and overall impacts of grazing exclusion on community stability. Table S6 Direct, indirect and total impacts of reseeded on community stability. [file ECE3-15-e70929-s001.docx]

**Reseeding native species promotes community stability by improving species diversity, niche and interspecific relationships in the desert steppe of northwest China**

Haixin Jiang^1,2,3,4^, Yao Zhou^1,2,3,4^, Wen Li^1,2,3,4^, Qi Lu^1,2,3,4^, Dongmei Xu^1,2,3,4^, Hongbin Ma^*1,2,3,4^, Xingzhong Ma^5^, Xiaowu Tian^6^

1. Research Center for Grassland and Animal Husbandry Engineering Technology of Ningxia Province, Ningxia University, 489 Helanshan West Road, Yinchuan 750021, Ningxia, China

2. Key Laboratory for Model Innovation in Forage Production Efficiency, Ministry of Agriculture and Rural Affairs, P.R.China, Ningxia University, 489 Helanshan West Road, Yinchuan 750021, Ningxia, China

3. Northern Yanchi Desert Steppe Observation and Research Station of Ningxia, Huamachi Town, Yanchi County, Wuzhong 751500, Ningxia, China

4. Ningxia University, College of Forestry and Praticulture, 489 Helanshan West Road, Yinchuan 750021, Ningxia, China

5. Ningxia Zhongning County Forestry and Grassland Bureau, Zhongning 755199 Ningxia, China

6. Ningxia Zhongwei State-owned Forestry Farm, Zhongwei 75000 Ningxia, China

Corresponding author: Hongbin Ma, Ningxia University, Yinchuan, China. E-mail: [ma_hb@nxu.edu.cn](mailto:ma_hb@nxu.edu.cn)

**FIGURE S1** Analysis of niche breadth and species importance value of reseeding grassland. a: Degradation grassland (0YEX(RS)); b: After 3 years of grassland grazing exclusion (3YEX); c: After 3 years of grassland reseeding (3YRS); d: After 6 years of grassland grazing exclusion (6YEX); e: After 6 years of grassland reseeding (6YRS). The same as below.

**TABLE S1** Composition of species and the life form of the plant community.

| No. | Abbreviation | Family name | Lifefrom |
| --- | --- | --- | --- |
| 1 | *Sc* | Polygonaceae | Annual herb |
| 2 | *As* | Compositae | Annual herb |
| 3 | *Sv* | Poaceae | Annual herb |
| 4 | *Co* | Polygonaceae | Annual herb |
| 5 | *Eh* | Euphorbiaceae | Annual herb |
| 6 | *Pm* | Poaceae | Annual herb |
| 7 | *Sr* | Amaranthaceae | Annual herb |
| 8 | *Am* | Poaceae | Perennialherb |
| 9 | *Gu* | Leguminosae | Perennialherb |
| 10 | *St* | Poaceae | Perennialherb |
| 11 | *Lb* | Plumbaginaceae | Perennialherb |
| 12 | *Pf* | Poaceae | Perennialherb |
| 13 | *Pb* | Rosaceae | Perennialherb |
| 14 | *Ca* | Convolvulaceae | Perennialherb |
| 15 | *Ls* | Poaceae | Perennialherb |
| 16 | *Al* | Leguminosae | Perennialherb |
| 17 | *Ee* | Euphorbiaceae | Perennialherb |
| 18 | *Sb* | Poaceae | Perennialherb |
| 19 | *Pt* | Polygalaceae | Perennialherb |
| 20 | *Aa* | Compositae | Perennialherb |
| 21 | *Ic* | Compositae | Perennialherb |
| 22 | *Sa* | Poaceae | Perennialherb |
| 23 | *Oi* | Compositae | Perennialherb |
| 24 | *Gv* | Leguminosae | Perennialherb |
| 25 | *Ms* | Poaceae | Perennialherb |
| 26 | *Ae* | Leguminosae | Perennialherb |
| 27 | *Lp* | Leguminosae | Subshrub |
| 28 | *Ox* | Leguminosae | Subshrub |
| 29 | *Ck* | Asclepiadaceae | Subshrub |
| 30 | *Oa* | Leguminosae | Subshrub |

Note : The full name corresponding to the abbreviation is as follows: *Sc*: *Salsola collina*; *As*: *Artemisia scoparia*; *Sv*: *Setaria viridis*; *Co*: *Corispermum tylocarpum*; *Eh*: *Euphorbia heyneana*; *Pm*: *Panicum miliaceum*; *Sr*: *Salsola tragus*; *Am*: *Agropyron mongolicum*; *Gu*: *Glycyrrhiza uralensis*; *St*: *Stipa breviflora*; *Lb*: *Limonium bicolor*; *Pf*: *Pennisetum flaccidum*; *Pb*: *Potentilla bifurca*; *Ca*: *Convolvulus ammannii*; *Ls*: *Leymus secalinus*; *Al*: *Astragalus laxmannii*; *Ee*: *Euphorbia esula*; *Sb*: *Cleistogenes serotina*; *Pt*: *Polygala tenuifolia*; *Aa*: *Aster altaicus*; *Ic*: *Ixeris chinensis*; *Sa*: *Stipa bungeana*; *Oi*: *Olgaea leucophylla*; *Gv*: *Gueldenstaedtia verna*; *Ms*: *Medicago sativa*; *Ae*: *Astragalus melilotoide*; *Lp*: *Lespedeza potaninii*; *Ox*: *Oxytropis racemosa*; *Ck*: *Cynanchum komarovii*; *Oa*: *Oxytropis aciphylla*.

**TABLE S2** Species importance value (IV) of reseeding grassland species in desert steppe. ‘-’ Represents no species present.

| No. | Species | 0YEX(RS) | 3YEX | 3YRS | 6YEX | 6YRS |
| --- | --- | --- | --- | --- | --- | --- |
| 1 | *Am* | 0.03±0.03 | 0.38±0.12 | 0.08±0.04 | 0.45±0.14 | 0.23±0.07 |
| 2 | *Lp* | - | 0.21±0.17 | 0.51±0.12 | 0.13±0.03 | 0.25±0.07 |
| 3 | *Sc* | - | - | 0.01±0.00 | 0.06±0.04 | 0.24±0.13 |
| 4 | *Gu* | 0.12±0.10 | 0.06±0.04 | 0.02±0.02 | 0.02±0.01 | 0.01±0.01 |
| 5 | *St* | - | - | - | 0.16±0.04 | 0.03±0.00 |
| 6 | *Ox* | - | - | - | 0.02±0.00 | - |
| 7 | *As* | - | 0.06±0.07 | 0.02±0.01 | 0.02±0.01 | 0.03±0.04 |
| 8 | *Lb* | - | - | - | 0.04±0.03 | 0.02±0.00 |
| 9 | *Pf* | 0.48±0.12 | 0.01±0.01 | 0.13±0.16 | 0.02±0.02 | 0.19±0.14 |
| 10 | *Pb* | - | - | - | 0.08±0.08 | 0.11±0.00 |
| 11 | *Ca* | - | - | - | 0.13±0.00 | - |
| 12 | *Sv* | - | - | 0.08±0.01 | - | - |
| 13 | *Ls* | 0.28±0.12 | 0.08±0.00 | - | - | 0.01±0.00 |
| 14 | *Al* | - | - | 0.14±0.13 | - | 0.02±0.01 |
| 15 | *Ee* | - | 0.01±0.00 | - | - | 0.01±0.00 |
| 16 | *Sb* | - | 0.03±0.02 | - | 0.05±0.04 | - |
| 17 | *Co* | - | - | - | 0.04±0.00 | - |
| 18 | *Pt* | - | - | - | 0.03±0.01 | - |
| 19 | *Aa* | - | 0.07±0.02 | - | 0.01±0.00 | - |
| 20 | *Oi* | - | 0.02±0.00 | - |  | - |
| 21 | *Ck* | - | 0.08±0.03 | - | 0.01±0.00 | - |
| 22 | *Eh* | - | - | 0.01±0.00 | - | - |
| 23 | *Ms* | - | - | 0.01±0.01 | - | - |
| 24 | *Ae* | - | - | 0.01±0.00 | - | - |
| 25 | *Pm* | - | - | 0.02±0.01 | - | - |
| 26 | *Ic* | - | 0.05±0.03 | - | - | - |
| 27 | *Sa* | - | 0.15±0.06 | - | - | - |
| 28 | *Gv* | - | 0.01±0.00 | - | - | - |
| 29 | *Oa* | - | 0.02±0.00 | - | - | - |
| 30 | *Sr* | 0.09±0.16 | - | - | - | - |

Note: 0YEX(RS): Degradation grassland; 3YEX: After 3 years of grassland grazing exclusion; 3YRS: After 3 years of grassland reseeding; 6YEX: After 6 years of grassland grazing exclusion; 6YRS: After 6 years of grassland reseeding. The same as below.

**TABLE S3** Levins niche widths of reseeding grassland species in the desert steppe. ‘-’ Represents no species present.

| No. | Specie | 0YEX(RS) | 3YEX | 3YRS | 6YEX | 6YRS |
| --- | --- | --- | --- | --- | --- | --- |
| 1 | *Am* | 2.00 | 4.82 | 4.30 | 4.51 | 4.61 |
| 2 | *Lp* | - | 2.24 | 4.55 | 4.70 | 3.56 |
| 3 | *Sc* | - | - | 1.00 | 3.27 | 3.78 |
| 4 | *St* | - | - | - | 2.97 | 1.00 |
| 5 | *As* | - | 1.89 | 2.57 | - | 2.17 |
| 6 | *Pf* | 2.86 | 1.00 | 2.14 | - | 3.11 |
| 7 | *Pb* | - | - | - | 1.82 | 1.00 |
| 8 | *Ls* | 2.03 | 1.00 | - | - | 2.95 |
| 9 | *Ee* | - | 1.00 | - | - | 1.00 |
| 10 | *Gu* | 1.72 | 3.77 | 2.79 | 1.80 | - |
| 11 | *Lb* | - | - | - | 2.57 | - |
| 12 | *Ca* | - | - | - | 1.00 | - |
| 13 | *Sb* | - | 3.64 | 2.80 | - | - |
| 14 | *Pt* | - | - | - | 4.59 | - |
| 15 | *Ck* | - | 2.67 | - | 1.00 | - |
| 16 | *Eh* | - | - | 1.00 | - | - |
| 17 | *Ms* | - | - | 2.09 | - | - |
| 18 | *Al* | - | - | 2.42 | - | - |
| 19 | *Sv* | - | - | 3.67 | - | - |
| 20 | *Pm* | - | - | 1.00 | - | - |
| 21 | *Aa* | - | 2.00 | - | - | - |
| 22 | *Ic* | - | 1.80 | - | - | - |
| 23 | *Sa* | - | 4.58 | - | - | - |
| 24 | *Oi* | - | 1.60 | - | - | - |
| 25 | *Oa* | - | 1.00 | - | - | - |
| 26 | *Sr* | 1.38 | - | - | - | - |

Note: 0YEX(RS): Degradation grassland; 3YEX: After 3 years of grassland grazing exclusion; 3YRS: After 3 years of grassland reseeding; 6YEX: After 6 years of grassland grazing exclusion; 6YRS: After 6 years of grassland reseeding. The same as below.

**TABLE S4** Table S5 Loads of Shannon-Weiner index and Margalef index on the first two principal components of principal component analysis (PCA). The first principal component is PC1; PC2, the second principal component.

| Model | PCA Index | Eigenvalue | Variance proportion |
| --- | --- | --- | --- |
| EX | Shannon-Wiener | 1.78 | 89.00% |
|  | Margalef | 0.22 | 11.00% |
| RS | Shannon-Wiener | 1.80 | 90.19% |
|  | Margalef | 0.20 | 9.81% |

**TABLE S5** Direct, indirect, and overall impacts of grazing exclusion on community stability.

| **From** | **To** | **Direct effect** | **Indirect effect** | **Total effect** |
| --- | --- | --- | --- | --- |
| **EX** | **community stability** | -0.12 | 0.66 | 0.54 |
| **EX** | **Niche width** |  | 0.17 | 0.17 |
| **EX** | **Niche overlap** |  | -0.16 | -0.16 |
| **EX** | **Interspecific association** |  | -0.12 | -0.12 |
| **EX** | **Pearson** |  | -0.03 | -0.03 |
| **EX** | **Diversity** | 0.70 |  | 0.70 |
| **Diversity** | **Niche width** | 0.24 |  | 0.24 |
| **Diversity** | **Niche overlap** | -0.42 | 0.19 | -0.23 |
| **Diversity** | **Interspecific association** |  | -0.17 | -0.17 |
| **Diversity** | **Pearson** |  | -0.05 | -0.05 |
| **Diversity** | **community stability** | 0.73 | 0.22 | 0.94 |
| **Niche width** | **Niche overlap** | 0.81 |  | 0.81 |
| **Niche width** | **Interspecific association** | 0.09 | 0.66 | 0.75 |
| **Niche width** | **Pearson** |  | 0.22 | 0.22 |
| **Niche width** | **community stability** | 0.41 | -0.42 | -0.01 |
| **Niche overlap** | **Interspecific association** | 0.81 |  | 0.81 |
| **Niche overlap** | **Pearson** |  | 0.24 | 0.24 |
| **Niche overlap** | **community stability** | -0.52 |  | -0.52 |
| **Interspecific association** | **Pearson** | 0.30 |  | 0.30 |

**TABLE S6** Direct, Indirect, and Total impacts of reseeded on community stability.

| **From** | **To** | **Direct effect** | **Indirect effect** | **Total effect** |
| --- | --- | --- | --- | --- |
| **RS** | **community stability** | 0.65 | 0.17 | 0.81 |
| **RS** | **Niche width** |  | 0.22 | 0.22 |
| **RS** | **Niche overlap** |  | -0.12 | -0.12 |
| **RS** | **Interspecific association** |  | -0.01 | -0.01 |
| **RS** | **Pearson** |  | 0.00 | 0.00 |
| **RS** | **Diversity** | 0.83 |  | 0.83 |
| **Diversity** | **Niche width** | 0.27 |  | 0.27 |
| **Diversity** | **Niche overlap** | -0.34 | 0.20 | -0.14 |
| **Diversity** | **Interspecific association** |  | -0.01 | -0.01 |
| **Diversity** | **Pearson** |  | 0.00 | 0.00 |
| **Diversity** | **community stability** | 0.13 | 0.07 | 0.20 |
| **Niche width** | **Niche overlap** | 0.74 |  | 0.74 |
| **Niche width** | **Interspecific association** | 0.29 | 0.44 | 0.73 |
| **Niche width** | **Pearson** |  | 0.11 | 0.11 |
| **Niche width** | **community stability** | 0.11 | -0.21 | -0.10 |
| **Niche overlap** | **Interspecific association** | 0.60 |  | 0.60 |
| **Niche overlap** | **Pearson** |  | 0.09 | 0.09 |
| **Niche overlap** | **community stability** | -0.28 |  | -0.28 |
| **Interspecific association** | **Pearson** | 0.14 |  | 0.14 |
